# Clinical and Imaging Profile of Patients with Cerebrotendinous Xanthomatosis – a Video Case Series from India

**DOI:** 10.5334/tohm.851

**Published:** 2024-03-06

**Authors:** Pavankumar Katragadda, Vikram V. Holla, Nitish Kamble, Jitender Saini, Ravi Yadav, Pramod Kumar Pal

**Affiliations:** 1 Department of Neurology National Institute of Mental Health and Neurosciences, Bengaluru, India; 2 Department of Neuroimaging & Interventional Radiology, National Institute of Mental Health and Neurosciences, Bengaluru, India

**Keywords:** Cerebrotendinous xanthomatosis, CYP27A1, cataract, ataxia, xanthoma

## Abstract

**Background:**

Cerebrotendinous xanthomatosis (CTX) is a rare autosomal recessive disorder caused by bi-allelic pathogenic variants in *CYP27A1* gene that results in the deposition of cholestanol in the eyes, tendons, soft tissues and nervous system leading to cataracts, xanthomas, and various neuropsychiatric manifestations. The aim of our study is to describe the clinical, radiological and genetic profile of patients with CTX.

**Methods:**

This is a retrospective chart review of patients with CTX diagnosed based on classical clinical and radiological findings. The available clinical details, and investigations, including imaging, electrophysiological, pathological and genetic data, were documented.

**Results:**

Five patients (4 males) were recruited in the study. The median age at presentation was 32 years (range: 21–66 years). Walking difficulty was the most common symptom at presentation. All patients had cataracts, tendon xanthomas, eye movement abnormalities, dysarthria, pyramidal signs, ataxia and gait abnormality. Dystonia was noted in three patients. Palatal tremor and parkinsonism were noted in one patient each. In MRI brain, dentate, and corticospinal tract involvement were the most frequent imaging findings. Bilateral hypertrophic olivary degeneration was noted in one patient and hot cross bun sign in two. Three patients underwent genetic testing and all had pathogenic variants confirming the diagnosis.

**Discussion:**

CTX is a rare treatable disorder. Apart from the usual neurological presentation with spastic-ataxia, it can present at a later age with parkinsonism. Typical patterns of imaging findings are helpful in early diagnosis which aids in the treatment to prevent the neurological sequelae of the disease.

## Introduction

Cerebrotendinous xanthomatosis (CTX) is a rare autosomal recessive disorder resulting from a defective enzyme in the bile acid synthesis pathway. It is caused by bi-allelic pathogenic variants in *CYP27A1* (2q35), which codes for sterol 27-hydroxylase. Reduction of the activity of this enzyme leads to increased formation and storage of abnormal lipid content with deposition of cholestanol in the eyes, tendons, soft tissues and brain and spinal cord, leading to early cataracts, tendon and soft tissue xanthomas, and various neuropsychiatric manifestations in the form of cognitive impairment, seizures, psychiatric disturbances, pyramidal and/or cerebellar signs, dystonia, atypical parkinsonism, and peripheral neuropathy. Symptoms can start early in life in the neonatal to infantile period with neonatal jaundice and chronic diarrhoea, which are often transient and self-limiting. The disease is potentially treatable with chenodeoxycholic acid. Early diagnosis and treatment are essential to prevent the neurological sequelae of the disease [1,2]. In this retrospective chart review, we aim to describe the clinical, radiological and genetic profile of five cases of CTX. In addition, we compared patients from our cohort to the previously reported large case series of patients with CTX.

## Materials and Methods

This is a retrospective chart review of patients with CTX who presented to the National Institute of Mental Health and Neurosciences, India, from 2017 to 2023. Patients were diagnosed based on the typical clinical and imaging features consistent with CTX, such as cataracts, tendon xanthomas, spasticity and ataxia with or without genetic diagnosis. The available demographics, clinical history, examination details, and investigations, including imaging, electrophysiological, pathological and genetic data, were documented. In addition, patient videos were also reviewed when available. The data were expressed using descriptive statistics. The patient videos were taken after written informed consent for video recording and publication.

## Results

### Clinical details

**Figure 1:**
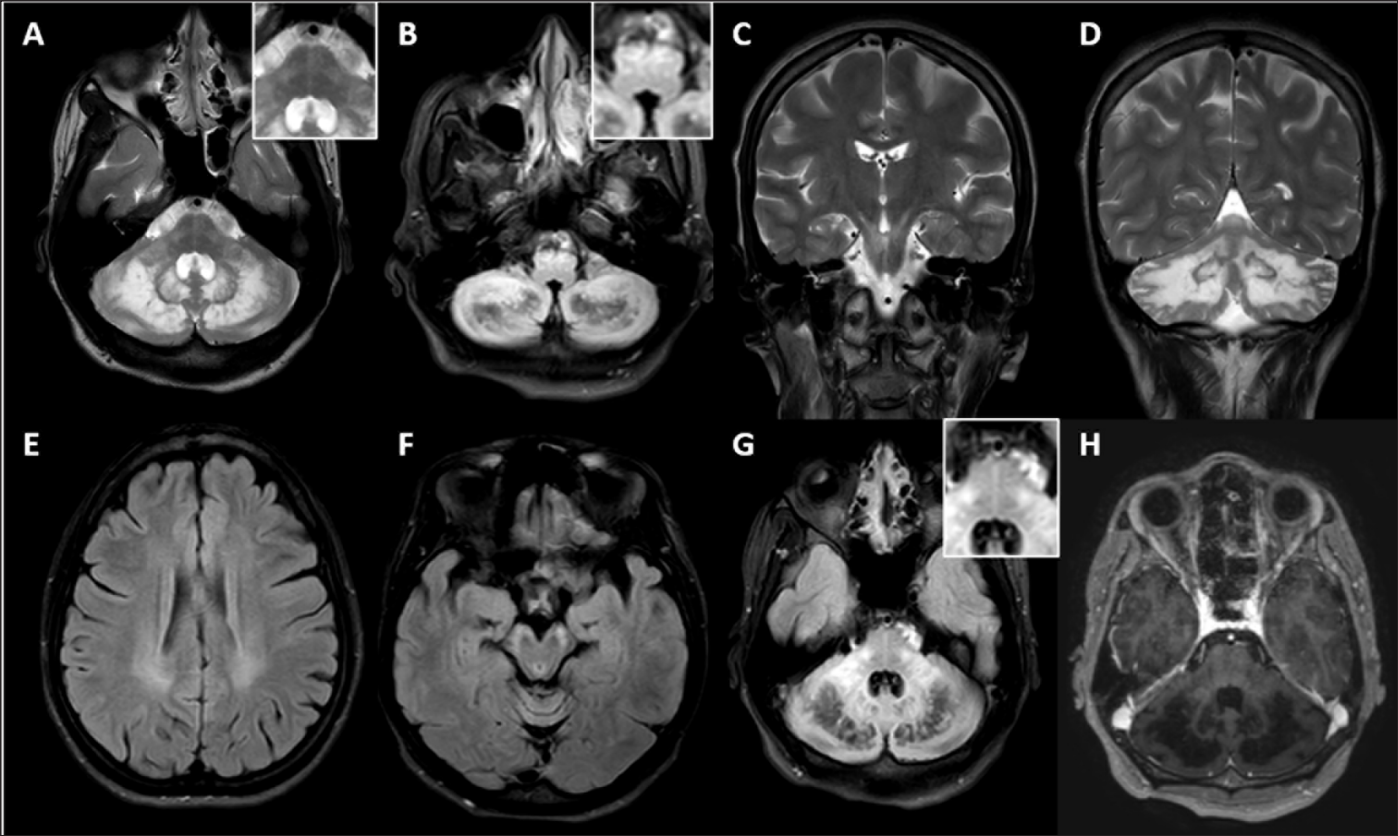
**MRI of Patient-1.** T2 **(A, C, D)**, FLAIR **(B, E-G)** and post contrast T1 **(H)** sequences of MRI brain of Patient-1 showing symmetric T2 hyperintense and FLAIR hypointense lesions along bilateral dentate and cerebellar white matter **(A, B, D, G)**, T2 and FLAIR hyperintensity of corticospinal tract along periventricular white matter **(E)**, posterior limb of internal capsule **(C)**, cerebral peduncles **(F)**, pons (A and H), inferior olives **(B)**, middle cerebellar peduncles (A and G) and rarefaction along cerebellar white matter (A and G). T2 hypointense rim noted around dentate nuclei **(D)**. Note the “Hot cross bun” appearance along the pons (A and G, inset) and olivary hyperintensity (B, inset). T1 post contrast image showed no enhancement **(H)**.

**Figure 2:**
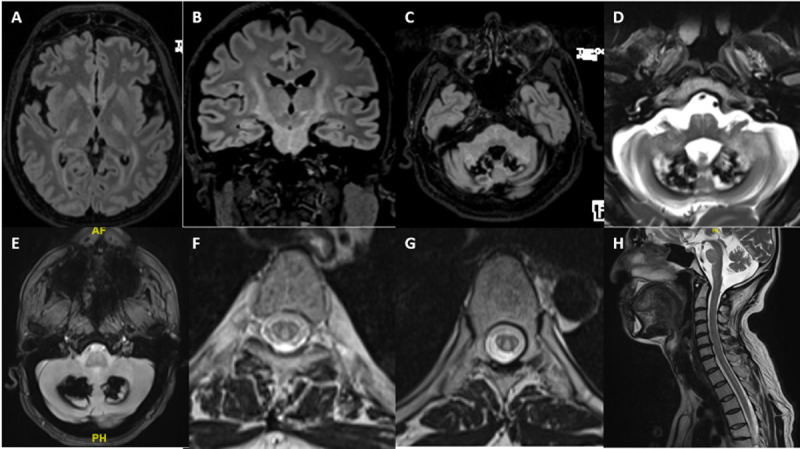
**MRI of Patient-2.** FLAIR axials **(A, C)**, coronal **(B)** and T2 axial **(D)** showing symmetric hyperintensities along periventricular white matter, corticospinal tracts, middle cerebellar peduncle, dentate and peri-dentate white matter, and along inferior olives. “Hot cross bun” sign can be seen within pons **(C)**. Susceptibility-weighted imaging **(E)** showing blooming in dentate nuclei. Long segment hyperintensity of dorsal and lateral columns noted in the axial **(F, G)** and sagittal **(H)** T2 sequences of MRI spine.

**Figure 3:**
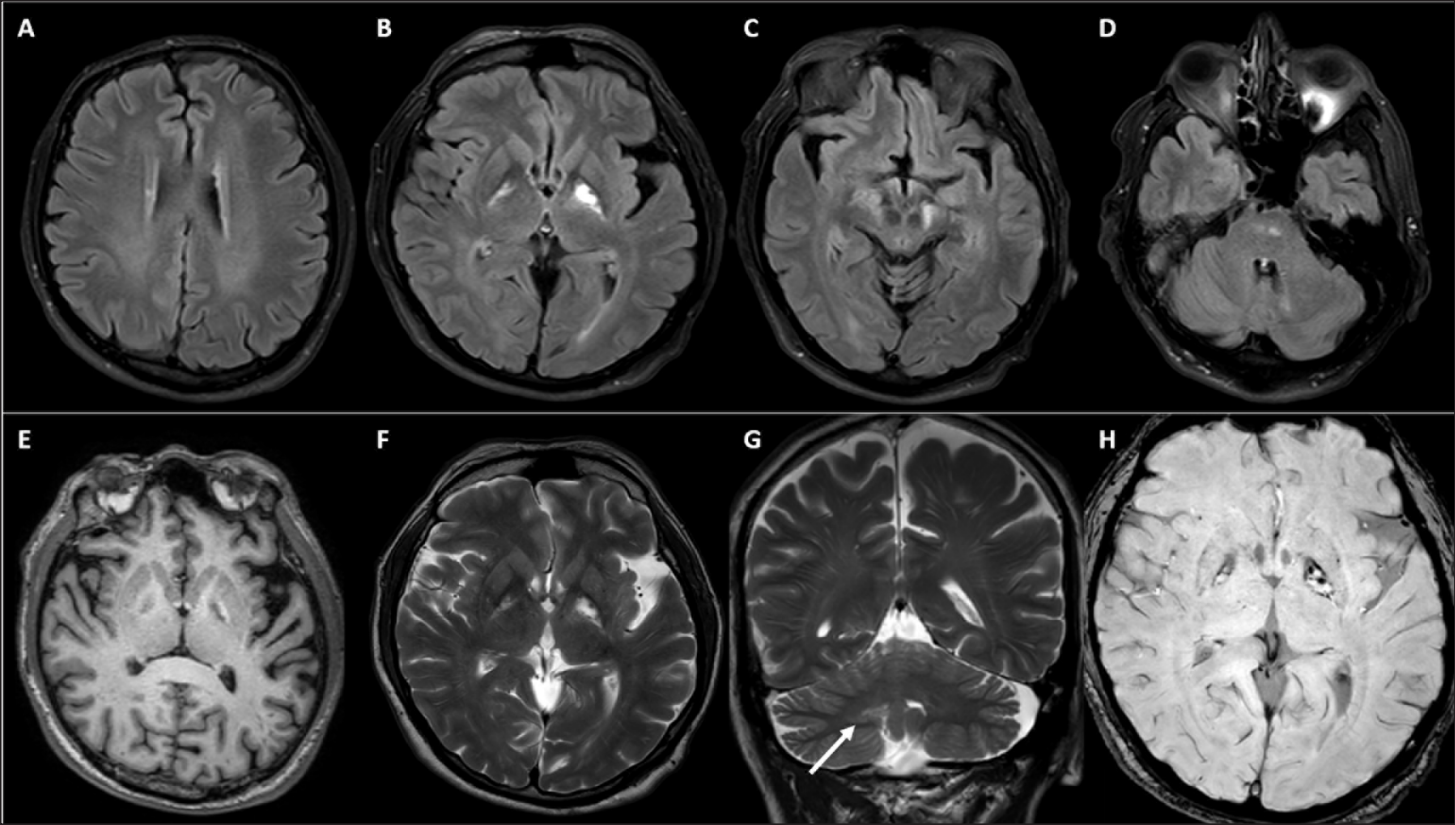
**MRI of Patient-3.** FLAIR axial images **(A-D)** showing hyperintensities along bilateral periventricular white matter and corticospinal tracts along internal capsules, midbrain and pons. Bilateral Globus pallidi appear hypointense on T1 **(E)** and hyperintense on T2 **(F)**. Bilateral dentate nuclei appear slightly hyperintense on coronal T2 images (G, white arrow). Susceptibility weighted imaging show blooming in left more than right globus pallidi lesions **(H)**.

**Figure 4:**
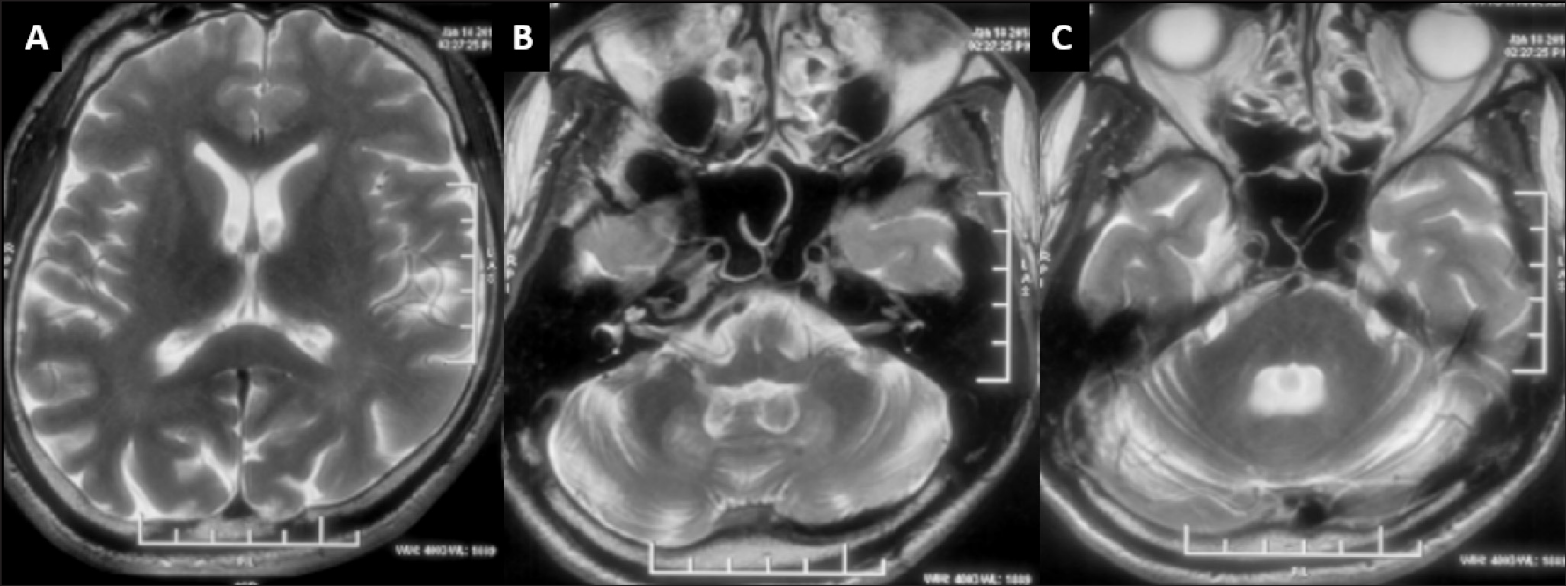
**MRI of Patient-4.** T2 axial images **(A-C)** showing mild diffuse cerebral and cerebellar atrophy with symmetric dentate nuclei hyperintensities.

**Figure 5:**
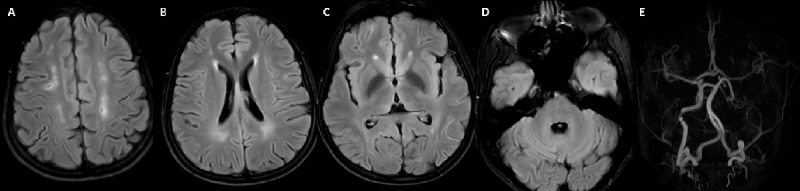
**MRI of Patient-5.** FLAIR axial sections **(A-D)** show chronic multiple watershed infracts in bilateral internal carotid artery territory (A) and subtle periventricular white matter signal changes **(B)**. Deep grey nuclei **(C)**, dentate and cerebellar white matter **(D)** appear normal. Time-of-flight MR angiography **(E)** show complete occlusion of the intracranial segment of the left internal carotid artery.

Five patients were recruited in the study based on classical clinical and radiological findings in all five patients, with genetic confirmation in three patients. The clinical vignettes of each case are provided in the supplementary document and the clinical and investigation details of all five patients are summarised in Table 1, MRI imaging findings are provided in Figures 1, 2, 3, 4, 5, and the clinical examination videos are provided as supplementary Videos 1, 2, 3, 4, 5.

**Table 1:** Demographic, clinical features, investigation findings and follow-up details of the cohort.


VARIABLES	PATENT-1	PATIENT-2	PATIENT-3	PATIENT-4	PATIENT-5

***Demographics***					

Age/AAO^@^/Gender	22y/17y/F	40y/30y/M	66y/64y/M	32y/28y/M	21y/20y/M

Family history/Consanguinity	–/–	–/+	+/–	–/–	+/–

***Symptoms***					

Presenting symptoms	Imbalance while walking, slurred speech, hand tremulousness	Walking difficulty, slurring of speech, pseudobulbar affect	Slurred speech, swallowing difficulty, pseudobulbar affect, walking difficulty, forgetfulness	Walking difficulty	Walking difficulty

Developmental delay	–	–	–	+	–

Intellectual disability	+	–	–	+	–

Seizures	–	+	–	+	–

Neonatal jaundice	–	–	–	–	–

Diarrhoea	–	–	–	–	–

Cataract (age at surgery)	+ (17 years)	+ (28 years)	+ (56 years)	+ (12 years)	+ (13 years)

Tendon xanthomas	+	+	+	+	+

***Examination findings***					

Eye movements	Square wave jerks Broken pursuits	Square wave jerks Broken pursuits	Square wave jerks Mild upgaze restriction	Dysmetric saccades Broken pursuits Nystagmus	Broken pursuits Nystagmus

Speech	Ataxic	Ataxic and spastic	Spastic	Spastic	Spastic

Spasticity	+	+	–	+	+

Weakness	–	–	–	B/L Toe grip weakness	–

Deep tendon reflexes	Brisk	Brisk	Brisk	Brisk	Brisk

Plantar	Flexor	Extensor	Flexor	Flexor	Extensor

Joint position	Normal	Normal	Normal	Impaired in great toes	Normal

Touch and Pinprick	Normal	Normal	Normal	Impaired below knee	Normal

Ataxia	+	+	+	+	+

Parkinsonism	–	–	+	–	–

Dystonia	+	+	–	+	–

Gait	Ataxic	Spastic	Ataxic	Spastic-ataxic	Spastic-ataxic

Additional findings	Palatal tremor, pes-cavus	Pes cavus	-	Pes-cavus	Pes-cavus, Feeble carotid and radial pulse

***Blood investigations***					

Lipid profile	Normal	Normal	Mild TG elevation	Normal	Normal

***Electrophysiology***					

Somatosensory evoked potential	Normal	Prolonged	Normal	Prolonged	Normal

Nerve conduction study	Normal	Normal	Normal	Sensorimotor axonal polyneuropathy	Normal

***Magnetic resonance imaging***					

Cerebellum	Dentate, peri-dentate, ICP	Dentate involvement	Normal	Atrophy with dentate signal changes	Normal

Brain stem	Signal changes along CST in the internal capsule, midbrain and pons, hypertrophic olivary degeneration	Signal changes along CST in the internal capsule, midbrain, pons and medulla	Signal changes along the CST in the internal capsule, midbrain and pons	Normal	Normal

Periventricular	Normal	Corona radiata	Frontal and parietal	Normal	Frontal and parietal

Angiography	Normal	Normal	Normal	Normal	Left CCA complete occlusion

Spine	Normal	Dorsolateral signal changes in cervical and dorsal cord	Normal	Normal	Not available

***Genetics^#^***					

Zygosity Variant Consequence	Not done	Homozygous c.379C>G p.Arg127Gly Missense	Comp-Het c.1184+1G>A/c.1537C>T NA/p.Arg513Cys Splice site/missense	Homozygous c.526del p.Asp176MetfsTer6 Frameshift truncation	Not done

***Treatment and follow-up***					

Treatment	UDCA: 600mg/d Atorvastatin:20mg/d Baclofen: 20mg/d LD/CD: 300/75mg/d	UDCA: 600mg/d Atorvastatin: 20mg/d Baclofen: 30 mg/d Amantadine: 200mg/d Valproate: 600 mg/d	UDCA: 600mg/d Atorvastatin: 20mg/d Baclofen: 20mg/d LD/CD: 400/100mg/d Escitalopram: 10mg/d	UDCA: 600mg/d Atorvastatin: 20mg/d Baclofen: 30 mg/d	UDCA: 600mg/d Atorvastatin: 40mg/d Baclofen: 20mg/d Aspirin: 150mg/d Clopidogrel: 75mg/d

Follow-up	Mild subjective improvement in gait at 3-months	Relentless progression over next 5-years to bedbound state	Worsening of overall deficits at 6-months Died after 1 year	Not available	Not available


+: Present; –: Absent, @: Age at onset of the neurological symptom, #: Reference transcript ID of *CYP27A1* gene – NM_000784.4. B/L: Bilateral, CCA: Common carotid artery, CST: Corticospinal tract, d: Day, F: Female, ICP: Inferior cerebellar peduncle, JPS: Joint position sense, LD/CD: Levodopa/carbidopa, LL: Lower Limb, M: Male, mg: Milligram, MRA: Magnetic resonance angiography, NA: Not applicable, NCS: Nerve conduction study, SSEP: Somatosensory evoked potential, TG: Triglycerides, UDCA: Ursodeoxycholic acid, UL: Upper limb.

**Video e1:** **Video of Patient-1.** Video demonstrating ataxic speech, facial dystonia, square wave jerks, saccadic intrusions, palatal tremor, impaired bilateral finger-nose test and heel-shin test, and ataxic gait. Bilateral Achilles tendon xanthomas can be seen in the segments demonstrating hell-shin test and while walking. The video was taken after written informed consent for video recording and publication.

**Video e2:** **Video of Patient-2.** Video demonstrating ataxic and spastic speech, square wave jerks, broken pursuit, impaired bilateral finger nose test, dysdiadokokinesia, spastic gait requiring support to walk, brisk deep tendon reflexes in all 4 limbs including ankle clonus, and bilateral extensor plantar response. Xanthomas can be seen over knee and Achilles tendon. The video was taken after written informed consent for video recording and publication.

**Video e3:** **Video of Patient-3.** Video demonstrating spastic speech, mild spooning of fingers, mild ataxic gait and postural instability. Tendon xanthoma can be seen in bilateral Achilles tendons. The video was taken after written informed consent for video recording and publication.

**Video e4:** **Video of Patient-4.** Video demonstrating spastic speech, gaze evoked nystagmus, impaired bilateral finger nose test, dysdiadokokinesia, impaired bilateral knee-heel-shin test, spastic-ataxic gait, and ankle clonus. Tendon xanthoma can be seen in bilateral Achilles tendons. The video was taken after written informed consent for video recording and publication.

**Video e5:** **Video of Patient-5.** Video demonstrating spastic speech, mild non-sustained gaze evoked nystagmus, impaired bilateral finger-nose test, spastic-ataxic gait, brisk ankle jerk and extensor plantar response. The video was taken after written informed consent for video recording and publication.

#### Clinical History

The median age at onset of neurological symptoms of the cohort was 28 years (Range: 17–64 years) and the median age at presentation was 32 years (Range: 21–66 years). Walking difficulty was the most common symptom observed in all five patients at the time of presentation. In two patients, it was the only presenting symptom, while in the other three patients, it was accompanied by additional symptoms such as dysarthria, dysphagia, pseudobulbar affect, tremulousness of hands, and forgetfulness. In the developmental and past history, global developmental delay with intellectual disability and seizure was noted in one patient, whereas intellectual disability and a history of seizure was noted in one patient each. Only one patient had parental consanguinity, and two others had a positive family history. Patient-3 had a similar illness in the father, with onset in the seventh decade (additional details not available), while Patient-5 had a sibling with a history of intellectual disability and seizures. All patients had undergone surgery for bilateral cataracts at various stages of their life.

#### Examination

All patients had Achilles tendon xanthoma, eye movement abnormalities, dysarthria, pyramidal signs, ataxia and gait abnormality. Among eye movement signs, saccadic and pursuits abnormalities were frequently noted in the form of square-wave jerks (three patients), dysmetric saccades (one patient) and broken pursuits (four patients). Additionally, two patients had horizontal gaze-evoked nystagmus. Dysarthria was more often spastic than ataxic. Among pyramidal signs, spastic tone was noted in four patients, hyperreflexia in all five patients, and extensor plantar response in two patients. Only one patient had sensory impairment and distal weakness in the form of impaired proprioception in the great toes, diminished touch and pain sensation below the knee, and bilateral toe grip weakness. In contrast to speech, gait abnormality had a more frequent ataxic component than a spastic component, with two patients having isolated ataxic component, spastic-ataxic in another two patients and isolated spastic component in the remaining patient.

Additional examination findings noted were bilateral pseudophakia in all five patients, post-surgical corneal opacity in the right eye in one, pes-cavus in four, right upper limb preaxial polysyndactyly in one, and feeble carotid and peripheral pulses in one patient.

#### Movement disorder phenomenology

Ataxia was the prominent movement disorder phenomenology noted in the form of eye movements abnormality in all five patients (square wave jerks, ill-sustained gaze-evoked nystagmus), ataxic component in speech in two patients, appendicular ataxia in four patients, and ataxic component of gait in four patients.

Apart from ataxia, the next frequent movement disorder phenomenology was dystonia noted in three patients, facial with mild cervical and bilateral finger dystonia in Patient-1 and mild bilateral finger dystonia in Patient-2 and 4. Palatal tremor without auditory click was noted in Patient-1. Parkinsonism in the form of hypomimia, appendicular bradykinesia and micrographia was noted in Patient-3. None of the patients had myoclonus or chorea.

### Investigations

#### Blood investigation

Among blood investigations, complete blood counts, renal function tests, liver function tests, thyroid function tests, random blood glucose, vitamin B12, folate, and homocysteine levels were normal in all five patients. Fasting lipid profile showed elevated triglyceride level only in Patient-4. Blood cholestanol level estimation could not be done in any of the patients as it is not commercially available in India.

#### Electrophysiology

Somatosensory evoked potential in the upper and lower limbs revealed prolonged latency in Patient-2 and 4, while it was normal in the other three patients. Nerve conduction study (NCS) revealed sensory-motor axonal neuropathy in the patient who had distal weakness with sensory impairment and was normal in the other four patients.

#### Imaging

MRI Brain was abnormal in all patients, while MRI spine was abnormal in one out of four patients in whom it was performed. In MRI brain, cerebellar-dentate and peri-dentate involvement (3 patients) and signal changes along the corticospinal tract in the brain stem and internal capsule (3 patients) were the most frequent imaging findings seen. Two patients had blooming on susceptibility-weighted imaging in the bilateral dentate and peri-dentate regions, while one other had blooming in the left internal capsule and the adjacent globus pallidus interna. Despite all patients having ataxia clinically, two patients had normal cerebellum on MRI. However, in these two patients, the ataxia was mild. Similarly, despite three patients having dystonia clinically, none of them had any involvement of basal ganglia. In one patient (Patient-3), where left globus pallidus interna was noted along with internal capsule involvement, there was no dystonia. Bilateral hypertrophic olivary degeneration along with inferior cerebellar peduncle involvement was noted in the patient who had palatal tremor without auditory clicks.

MR angiography was performed in Patient-5 in view of clinical findings of feeble carotid pulses and revealed complete occlusion of the left internal carotid artery. MRI spine was abnormal in Patient-2 with longitudinally extensive, confluent, signal changes along the dorsolateral white matter tracts in cervical and dorsal spine regions.

#### Genetic testing

Clinical exome sequencing was performed in three patients and pathogenic variants were identified in all three of them in *CYP27A1* gene; a pathogenic homozygous missense variant (NM_000784.4:c.379C>G;p.Arg1276Gly) in Patient-2, pathogenic compound heterozygous splice site variant (NM_000784.4:c.1184+1G>A) and a missense variant (NM_000784.4:c.1537C>T;p.Arg513Cys) in Patient-3, and a pathogenic homozygous single base pair deletion variant (NM_000784.4:c.526del;p.Asp176MetfsTer6) in Patient-4. All the four variants have been reported previously. Sanger sequencing could not be done in the parents of patient-3 to establish the trans configuration of the variants due to the non-availability of the parents’ samples. Investigation details are provided in Table 1, clinical vignettes of each patient are provided in the supplementary document, and radiological images are provided in Figures 1, 2, 3, 4, 5.

### Treatment and follow-up

None of the patients received chenodeoxycholic acid (CDCA) as the compound is unavailable in our country. All patients received 600mg/day dose of ursodeoxycholic acid and 40 mg/day dose of atorvastatin. In addition, symptomatic medications were also initiated in the form of baclofen for spasticity in all five patients, levodopa/carbidopa combination for palatal tremor in Patient-1 and parkinsonism in Patient-3, amantadine for ataxia and valproate for seizures in Patient-2, and escitalopram for pseudobulbar affect in Patient-3. Patient-5 also received dual antiplatelets in view of extensive vascular disease.

Follow-up details are available for three patients. Patient-1 had mild subjective improvement in gait at 3-months follow-up. Patient-2, followed up over 5 years and was noted to have relentless progression in all his signs and symptoms with severe dysarthria, worsening of pyramidal and cerebellar involvement, requiring two persons support to walk and dependant for all daily activities. Patient-3 also progressed relentlessly and succumbed to his illness after one year due to aspiration pneumonia secondary to severe bulbar involvement. Follow-up details were not available for Patient-4 and 5.

We compared patients from our cohort to previously reported large case series of patients with CTX (Table 2) [2,3,4,5,6,7,8]. None of our patients had neonatal jaundice and diarrhoea, while diarrhoea was reported in about 20–90% of patients in other cohorts. Mignarri et al. reported neonatal jaundice in 15% of their patients [4]. Intellectual disability, cognitive impairment, and polyneuropathy were less frequent in our study compared to other case series. The rest of the demographic and clinical features of our patients were comparable to other larger case series. Among the movement disorders, cerebellar signs were seen in 36% to 89% patients in these studies [4,6]. Parkinsonism was noted in 9–20% in various studies compared to 20% in our cohort [3,4]. In a case series with review of previously reported cases of CTX with movement disorders, Stelten et al observed that parkinsonism (52%) was second most common movement disorders after ataxia followed by dystonia, myoclonus, and postural tremor [9].

**Table 2:** Comparison of Demographic, Clinical, and Radiological profiles with other cerebrotendinous xanthomatosis series.


	OUR COHORT	DUELL ET AL (2018) [2]	SEKIJIMA ET AL (2018) [3]	MIGNARRI ET AL (2014) [4]	PILO DE LA FUENTE ET AL (2011) [5]	LEE ET AL (2001) [6]	VERRIPS ET AL (2000) [7]	BERGINER ET AL (1984) [8]	STELTEN ET AL (2019) [9]

**Number of patients**	5 (1F, 4M)	43	40 (20M, 20F)	55 (28F, 27M)	25 (15F,10M)	19 (12F,7M)	54	17 (12F, 5M)	62 (29F/30M)

**Age at onset (years)**	19.2 ± 16.14	32	24.5 ± 13.6	9.5 ± 9	19.2 ± 11.2	Not reported	14 ± 11.6	Not reported	Not reported

**Age at Diagnosis (Years)**	36.2 ± 18.3	Not reported	41 ± 11.6	35.5 ± 11.8	38 ± 10.7	Not reported	33.5 ± 10.9	Not reported	35 ± 11

**Consanguineous parents**	1/5 (20%)	Not reported	7/40 (17.5%)	13/39 (33%)	Not reported	0/12 (0%)	2/32 (6%)	Not reported	Not reported

**Tendon Xanthomas**	5/5 (100%)	33/43 (77%)	38/40 (95%)	43/55 (78%)	14/25 (56%)	19/19 (100%)	27/54 (50%)	15/17 (88%)	47 (76%)

**Cataract**	5/5 (100%)	30/43 (70%)	24/40 (60%)	49/55 (89%)	23/25 (92%)	14/19 (74%)	52/54 (96%)	12/17 (71%)	51 (82%)

**Chronic Diarrhea**	0/5 (0%)	23/43 (53%)	8/40 (20%)	22/55 (40%)	23/25 (92%)	Not reported	19/54 (35%)	Not reported	19 (31%)

**Neonatal Jaundice**	0/5 (0%)	Not reported	Not reported	8/55 (15%)	Not reported	Not reported	Not reported	Not reported	Not reported

**ID/Cognitive impairment**	2/5 (40%)	32/43 (74%)	28/40 (70%)	33/55 (60%)	12/25 (48%)	Not reported	33/54 (61%)	Not reported	54 (87%)

**Behavioral Disturbances**	0/5 (0%)	Not reported	Not reported	24/55 (44%)	14/25 (56%)	Not reported	Not reported	Not reported	23 (37%)

**Epilepsy**	2/5 (40%)	Not reported	4/40 (10%)	18/55 (33%)	8/25 (32%)	Not reported	14/54 (26%)	Not reported	18 (29%)

**Pyramidal signs**	5/5 (100%)	Not reported	26/40 (65%)	35/55 (64%)	23/25 (92%)	17/19(89%)	39/54 (72%)	17/17 (100%)	46 (74%)

**Cerebellar signs**	4/5 (80%)	Not reported	17/40 (42.5%)	20/55 (36%)	20/24 (83%)	17/19(89%)	35/54 (65%)	13/17 (76%)	42 (68%)

**Parkinsonism**	1/5 (20%)	Not reported	8/40 (20%)^*^	5/55 (9%)	Not reported	Not reported	Not reported	Not reported	32 (52%)^@^

**Polyneuropathy**	1/5 (20%)	Not reported	5/40 (12.5%)	21/30 (70%)	12/18 (67%)	12/19 (63%)	19/24 (79%)	7/17 (41%)	28 (45%)

**MRI-Dentate involvement**	3/5 (60%)	Not reported	25/40 (62.5%)	20/26 (77%)	14/23 (61%)	Not reported	28/34 (82%)	Not reported	38/47 (81%)

**High Serum Cholestanol**	Not Done	43/43 (100%)	40/40 (100%)	37/37 (100%)	14/14 (100%)	19/19 (100%)	43/43 (100%)	17/17 (100%)	Not reported


F: Female; ID: Intellectual disability; M: Male; MRI: Magnetic resonance imaging. *: Parkinsonism/dystonia. @: Dystonia-19 (31%), myoclonus-11 (18%), tremor-6 (10%), unspecified extrapyramidal signs-10 (16%).

## Discussion

Cerebrotendinous xanthomatosis is a rare treatable neurological disorder with a wide spectrum of neurological and non-neurological manifestations with symptom onset ranging from early infancy to late adulthood. Early diagnosis is imperative as stabilisation and improvement in disease manifestations are more likely if the treatment starts early. Clinical features of CTX often follow a temporal profile [10]. The earliest manifestation is the transient and self-limiting neonatal jaundice due to elevated conjugated hyperbilirubinemia and chronic unexplained infantile diarrhoea, both seen in around 75% of the patients. Bilateral cataracts seen in about 85% of the patients start developing in the childhood to adolescence age group. Tendinous xanthomas often develop after cataracts and can be seen in around 70% of cases [4]. Achilles tendon is the most commonly involved site. The other sites are tibial tuberosity, triceps, and finger tendons. Neurological dysfunction is almost universal, with onset usually around late adolescence and early adulthood similar to xanthomas. Pyramidal signs and cerebellar dysfunction are the most frequent neurological abnormalities occurring in more than two-thirds of the cases. Intellectual disability is frequently seen and observed from the first decade itself.

Similar observations were also noted in our cohort. Tendon xanthomas, cataracts, pyramidal signs and ataxia were noted in all five patients and intellectual disability was noted in two patients. However, in contrast to previous studies, none of our patients reported either neonatal jaundice or infantile diarrhoea. This may be due to the recall bias as all the patients presented after late second decade. In a study done by Friedman et al. on the prevalence of CTX in patients with idiopathic bilateral cataracts with onset in 1^st^ and 2^nd^ decade, 1.8% of patients were diagnosed as having CTX [11]. Juvenile-onset idiopathic bilateral cataracts may be helpful as a screening marker for CTX. Mignarri et al. devised a suspicion index for CTX after reviewing five large series of CTX patients, and they found that tendon xanthomas and juvenile-onset cataracts are strong predictors of CTX, with tendon xanthomas being more specific for CTX [4]. Even though all patients in our cohort exhibited bilateral cataracts and tendon xanthomas before experiencing neurological symptoms, none underwent evaluation for CTX until neurological manifestations started. The early identification and treatment of CTX can be significantly facilitated by raising awareness among ophthalmologists and paediatricians.

Although not common, movement disorders phenomenology apart from ataxia can be observed in patients with CTX. Stelten et al., in their study of seven patients, along with a review of previously reported cases of CTX with movement disorders, observed that parkinsonism was the second most common movement disorder after ataxia, followed by dystonia, myoclonus, and tremor [9]. Mixed movement disorders were observed in 23% of patients. While in 18% of the cases, a movement disorder was the initial symptom, in the others they were usually part of a complex clinical picture rather than a prominent symptom. Parkinsonism was observed to be more often asymmetric with bradykinesia and rigidity with or without rest tremor and postural instability. Dystonia was often focal or multifocal with frequent involvement of facial, oromandibular, cervical and upper limbs. There are two reports of palatal tremor or myoclonus involving pharyngeal, laryngeal or lingual movements [12,13]. In the case reported by Rossi et al, the patient had progressive ataxia and palatal tremor phenotype of CTX without xanthoma or cataract. In addition, there were a few reports of postural upper limb tremor and distal predominant myoclonus in CTX [14,15,16]. Myoclonus in CTX has been reported to be of subcortical origin based on the electrophysiological findings and may resemble postural tremor to the untrained eyes [14,15]. In our cohort, dystonia was more frequent, seen in three patients, compared to parkinsonism seen in one patient. The distribution of dystonia involving facial, cervical and upper limbs was in line with the previous reports. Apart from these, one patient had palatal tremor without auditory clicks. There were no patients with chorea or myoclonus in our cohort.

Imaging provides insight towards understanding the pathophysiology behind the observed movement disorder phenomenology in patients with CTX. Dentate nucleus and cerebellar abnormality can explain ataxia in the majority but not in all. Two patients in our cohort had ataxia albeit mild, without any radiological cerebellar abnormalities. In such cases, involvement of the cerebellar outflow tract and its connections in the brainstem and cerebrum can explain the ataxia. Contrary to the expectation, in the study by Stelten et al, basal ganglia involvement was observed in only a small number of CTX patients exhibiting movement disorder symptoms, predominantly in patients with parkinsonism [9]. Even in patients with parkinsonism, less than a quarter of patients had involvement of substantia nigra, globus pallidus, and/or striatum on routine MRI. However, presynaptic dopaminergic dysfunction was noted in 9 out of 10 patients with parkinsonism, suggesting nigral dysfunction as one of the main driving factors. In contrast, post-synaptic dysfunction was reported in only 1 out of 3 patients studied. One patient in our cohort had mild parkinsonism with involvement of the left globus pallidus along with the adjacent internal capsule. However, we did not perform tracer imaging on this patient.

Basal ganglia abnormality on MRI was seldom noted in patients with movement disorders other than parkinsonism such as dystonia. This may suggest pathology involving regions other than basal ganglia are in play. In this regard, abnormal cerebellar outflow due to dentate abnormality or dysfunction of networks involved in sensorimotor integration due to the involvement of various tracts could play a role in the pathogenesis of dystonia in CTX. Even in our study, none of the three patients with dystonia had basal ganglia involvement, while all had cerebellar abnormality. Palatal tremor or palatal myoclonus reported in CTX, may be is secondary to the involvement of the olivary nucleus, resulting in disruption of the dentato-rubro-olivary circuit [12]. Apart from the bilateral olivary hyperintensity without hypertrophy noted in our case, olivary abnormality has been reported in two previously reported cases of CTX with palatal tremor; thinning of olives on post-mortem study by Philippart et al [17]. and olivary hyperintensity without hypertrophy on MRI by Rossi et al [12].

Barkhof et al. reported imaging findings in 24 patients of CTX [18]. In their study, typical hyperintense lesions were seen on T2-weighted images in the dentate nucleus (in 79% of patients), globus pallidus, substantia nigra, and inferior olive and extended into the adjacent white matter as the disease progressed. Hypointensity was found on T2-weighted images in the dentate nucleus and was related to hemosiderin deposition and calcifications. Spinal cord MR imaging revealed increased signal intensity in the lateral and dorsal columns on T2-weighted images. Periventricular white matter changes with premature internal carotid atherosclerosis seen in a patient in this study highlights the fact that CTX patients can have premature cerebrovascular atherosclerosis as isolated imaging findings [19]. An interesting finding of the pontine hot-Cross bun sign was seen in one of our patients, and there are only two previous reports of this sign in patients with CTX [20,21].

CTX is caused by biallelic pathogenic variants in *CYP27A1* via a loss of function mechanism. Many of the reported pathogenic variants involve splice sites. They are predicted to affect mRNA stability or lead to abnormal mRNA formation with translation products devoid of an adrenodoxin-binding region and/or the heme-binding site, which is important for enzyme activity. Other pathogenic variants are predicted to result in truncated peptides devoid of function [6,7]. In our cohort, genetic testing was done in three patients; all had previously reported pathogenic variants in the *CYP27A1* gene.

Chenodeoxycholic acid (CDCA) is considered the standard of care for treating patients with CTX. Oral administration of CDCA has been evaluated by Berginer et al. in their cohort of 17 patients with CTX and was found to be effective in correcting the biochemical abnormalities and improving the clinical symptoms [8]. CDCA inhibits the cholesterol 7α-hydroxylase enzyme and provides negative feedback on cholesterol biosynthesis, which reduces abnormal bile acid synthesis. This is mediated through a pathway involving the nuclear receptor FXR, which inhibits CYP7A1 [22,23,24]. CDCA is effective in improving both the biochemical abnormalities and clinical features of CTX [22]. However, advanced symptoms present for many years are unlikely to show significant improvement. CDCA treatment does not improve tendon xanthomas or cataracts. However, it can stabilise and improve cognitive and neurological symptoms, such as pyramidal tract signs and cerebellar deficits [8]. CDCA treatment is more effective in reducing neurologic manifestations when started early in the disease course. CTX patients treated with CDCA after the age of 25 years had worse outcomes and were significantly more limited in ambulation and cognition [25]. Unfortunately, none of the patients in our cohort received CDCA due to its unavailability in our country. Two of our patients had relentless progression, and one of them succumbed to the illness. As they were not treated with CDCA, it is difficult to comment on whether advanced disease or inadequate treatment led to this poor neurological outcome.

Limitations of this study include small sample size and lack of genetic confirmation in two patients. In the two patients without genetic confirmation, both had classical clinical and radiological features making diagnosis other than CTX very unlikely. However, biochemical or genetic testing is essential to confirm the diagnosis. Despite diagnosing this rare but treatable neurological disorder, we could not offer the medication due to its unavailability. Efforts are needed to make the access to these treatment in rare diseases worldwide as there are a very few rare diseases that has specific medications and often it need to be initiated at an early stage.

## Conclusion

Cerebrotendinous xanthomatosis is a rare, treatable genetic disorder of bile acid and cholesterol metabolism. Apart from the usual neurological presentation with ataxia and spasticity, it can present at a later age with Parkinsonism. The presence of early cataracts and tendon xanthomas are important clues for diagnosis. Typical patterns of imaging findings are helpful in diagnosis. Premature cerebrovascular atherosclerosis and dorsolateral spinal cord involvement can be seen rarely. Early diagnosis and treatment are essential to prevent the neurological sequelae of the disease.
